# Estimating the reproductive number and the outbreak size of COVID-19 in Korea

**DOI:** 10.4178/epih.e2020011

**Published:** 2020-03-12

**Authors:** Sunhwa Choi, Moran Ki

**Affiliations:** Department of Cancer Control and Population Health, Graduate School of Cancer Science and Policy, National Cancer Center, Goyang, Korea

**Keywords:** COVID-19, Epidemiology, Mathematical model, Outbreak, Reproduction number, Korea

## Abstract

**OBJECTIVES:**

Since the first novel coronavirus disease 2019 (COVID-19) patient in Korea was diagnosed on January 20, 2020, 30 patients were diagnosed until February 17, 2020. However, 5,298 additional patients were confirmed until March 4, 2020. Therefore, our objective was to estimate the reproduction number (*R*) and evaluate the effectiveness of preventive measures.

**METHODS:**

A COVID-19 transmission model (SEIHR) was established to suit the Korean outbreak. The number of daily confirmed cases in Daegu and North Gyeongsang Province (NGP), the main area of outbreak, was used. The first patient’ symptom onset date in the Daegu/NGP outbreak was assumed as January 22, 2020. The *R* according to the start date of the effect of preventive measures was estimated.

**RESULTS:**

The estimated *R* in Hubei Province, China, was 4.0281, whereas the estimated initial *R* in Korea was 0.555, but later in Daegu/NGP, the value was between 3.472 and 3.543. When the transmission period decreases from 4-day to 2-day, the outbreak ends early, but the peak of the epidemic increases, and the total number of patients does not change greatly. It was found that, if transmission rate decreases, the outbreak ends early, and the size of the peak and the total number of patients also decreases.

**CONCLUSIONS:**

To end the COVID-19 epidemic, efforts to reduce the spread of the virus, such as social distancing and wearing masks, are absolutely crucial with the participation of the public, along with the policy of reducing the transmission period by finding and isolating patients as quickly as possible through the efforts of the quarantine authorities.

## INTRODUCTION

At the end of December 2019, China reported a case of pneumonia with unknown cause. The disease was subsequently found to be caused by a novel coronavirus and the World Health Organization (WHO) had named the new disease novel coronavirus disease 2019 (COVID-19). As of March 8, 2020, there are approximately 100,000 infected people worldwide and more than 7,000 cases have been confirmed in Korea [1,2].

Starting with the first confirmed case on January 20, 2020, thirty cases were confirmed in Korea over a period of about 30 days until February 17, 2020. In most cases, people were infected while abroad and then spread the infection domestically upon return, or the disease was spread domestically by these index patients. However, starting with patient #31 on February 18, 2020, there has been an unprecedented rapid and large-scale epidemic in Daegu and North Gyeongsang Province (NGP). In the process of tracking those who came in contact with patient #31 on February 18, 2020, it was found that patient 331 had visited the Shincheonji Church in Daegu and had attended the service in Daegu on February 9, 2020 and 16, 2020, suggesting the possibility of transmission to other attendees. On February 20, 2020, a complete inspection began on 1,000 people who attend the Shincheonji Daegu Church. In addition, upon discovering that patient #31 had also visited Cheongdo in NGP in early February, the investigation on the possibility of connection with Daenam Hospital in Cheongdo also began. Subsequently, Daegu and NGP saw a rapid increase in confirmed COVID-19 cases, leading to 5,298 additionally confirmed cases since the diagnosis of patient #31 over a period of 15 days until March 4, 2020. The quarantine authorities actively responded by raising the risk alert for infectious disease to the serious level on February 23, 2020. Therefore, it is necessary to estimate the scale of the epidemic by estimating the initial infection reproduction number (*R*) for COVID-19 in Korea as well as the *R*s for Daegu and NGP, in order to provide information critical in determining current and future intervention policies.

## MATERIALS AND METHODS

### Data

To estimate the parameters for the mathematical model, the press releases from the Korea Centers for Disease Control and Prevention (KCDC) were analyzed and the cases confirmed between January 20, 2020 and March 4, 2020 were used [2]. For the total population of Daegu, NGP, and Korea, the 2019 data from National Statistics portal were used [3]. For the number of daily confirmed cases in China, the official reports from the Chinese government were used.

### Mathematical model

**Figure 1.**
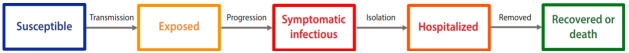
Deterministic SEIHR model for coronavirus disease 2019.

The transmission model for COVID-19 infection is a deterministic SEIHR model. In this model, the population is divided into 5 groups including susceptible (S), exposed (E), symptomatic infectious (I), hospitalized (H), recovered or death (R) (Figure 1).

The following assumptions are made in this model:

(1) Births and natural deaths in the population are not taken into account; (2) Infections during latency and asymptomatic infections are not taken into account; (3) Patient cannot spread disease while in isolation treatment after confirmed diagnosis; and (4) Recovered patients do not get re-infected.

Those in the S group are infected via contact with those in the I group and move to the E group. The mathematical model of COVID-19 infection transmission is expressed as follows. The total population (N) is equal to the sum of the populations in each group. Here, the change in the population of each group over time is as shown in the equation below (Equation 1).

Model constant *β* indicates the rate of infection transmission; and those who are exposed show symptoms after a certain period of time, turning into infectious patients who can transmit the disease. The constant *κ* refers to the progression of symptoms of COVID-19 while 1/*κ* refers to the mean latency period of COVID-19. Infected patients who express symptoms are confirmed positive after a certain period of time and are isolated. The rate of confirmation and isolation after symptom onset in I patients is denoted by *α*, and 1/*α* is the mean period from symptom onset to confirmation. This is the period during which the infection can spread. The constant *γ* represents the recovery rate of the infected patients in isolation and 1/*γ* represents the mean period of isolation until recovery.

Equation 1. Differential equation for population changes over time in deterministic SEIHR model for coronavirus disease 2019. $\frac{dS}{dt}=-\beta \frac{1}{N}S,\;\frac{dE}{dt}=\beta \frac{1}{N}S-\kappa E,\;\frac{dI}{dt}=\kappa E-\alpha I,\;\frac{dH}{dt}=\alpha I-\gamma H,\;\frac{dR}{dt}=\gamma H,\;N=\;S+E+I+H+R$

The definitions and values of the parameters in the COVID-19 infection transmission model are shown in Table 1. For the progression rate (*κ*), isolation rate (*α*), and recovery rate (*γ*), the results of data analysis on infection latency period (4 days), mean period since symptom onset to confirmation (4 days), and mean isolation period until recovery (14 days) from the early confirmed cases in Korea were referenced, respectively, according to each definition [4].

For transmission rate (*β*), it was estimated to minimize the square of the difference in the estimated number of confirmed cases in data (*x*) and in the model (*αI*) using the least square fitting method on the daily cumulative confirmed cases data and the cumulative number of confirmed cases on the corresponding date in the model. In other words, the *β* that minimizes $\sum_{i}{\left(cumulative{\left[x\right]}_{i}-cumulative{\left[\alpha I\right]}_{i}\right)}^{2}$ was estimated using the “lsqcurvefit” package by Matlab (Table 1).

### Reproduction number

The *R* refers to the average number of secondary infected persons by one primary infected patient during the infectious period. If *R* is greater than 1, the number of infected patients increases; if *R* is less than 1, the number of infected patients decreases, and the disease will die out. When *R*=1, it suggests that the infectious disease has become an endemic and a certain number of patients are continuously present within a community.

*R* is determined by the probability of the spread of infection upon contact with an infected person, the number of contacts with the infected person, and the duration for which the infected person can spread the infection. Here, *β* can also be described as the probability of transmission of the infection multiplied by the contact level. The *R* of COVID-19 calculated from the COVID-19 infection mathematical model in this study is as follows. Here, β is the transmission rate and 1/*α* is the infection transmission period.

$$R=\frac{\beta }{\alpha }$$

In order to estimate the *R* at the initial stage of COVID-19 transmission in Hubei, China, the data from daily confirmed cases between December 29, 2019 and February 3, 2020 were used.

In estimating the *R* at the initial stage of COVID-19 transmission in Korea, the data from the date of symptom onset in 30 confirmed cases between January 20, 2020 and February 17, 2020 were used. In addition, the cases of overseas infection were separated from domestically infected cases to apply the overseas inflow function to the model. For those who were infected while abroad, they were assumed to have entered the I group on the day of symptom onset in the COVID-19 transmission model (Figure 1). If those who were infected while abroad returned to Korea after symptom onset, then they were assumed to have moved to Group *I* on the date of entry into Korea.

For Daegu and NGP in Korea, it was assumed that 1 infected person with symptoms entered the region on January 22, 2020, the day before Wuhan, China was closed off. Based on the daily confirmed cases in Daegu and NGP between February 18, 2020 and March 4, 2020, *R* was estimated. As *R* changed over time, the change in *R* was reviewed by increasing the data fitting period by 1 day based on the period from February 18, 2020 until February 24, 2020.

## RESULTS

### COVID-19 early stage epidemic model for Hubei, China

The *β* for Hubei Province was estimated as 0.806 (95% confidence interval [CI], 0.802 to 0.810), and the *R* was estimated to be 4.028 (95% CI, 4.010 to 4.046).

**Figure 2.**
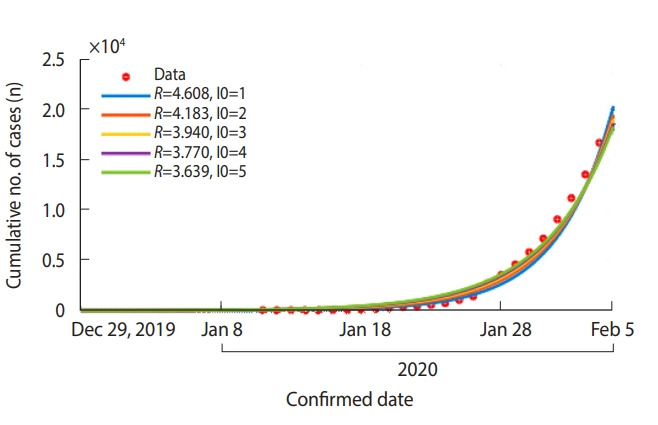
Estimated reproductive number (*R*) by daily cumulative reported patients with coronavirus disease 2019 in Hubei Province, China. The number of infected patients (I0) on December 29, 2019 was assumed as 1 to 5. Cumulative number of cases (red dots) and model fitting curves (colored lines).

In addition, as the start date of the epidemic in Hubei is unclear, it could be assumed that there were up to five infected patients (I0) on December 29, 2019, rather than one patient, which changes *R* from 3.639 (when initial number of patients is 5) to 4.608 (when initial number of patients is 1) (Figure 2).

### COVID-19 early stage epidemic model for Korea

**Figure 3.**
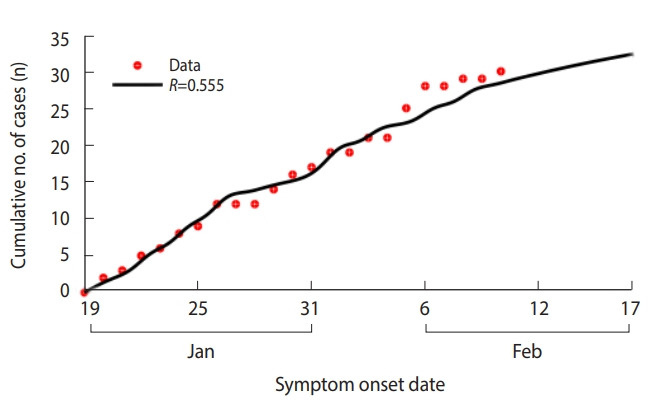
Estimated reproductive number (*R*) by daily cumulative symptom onset patients with coronavirus disease 2019 in Korea from January 20, 2020 to February 17, 2020. Cumulative number of cases (red dots) and model fitting curves (colored lines).

When the model was applied to the first 30 cases in Korea, the COVID-19 *β* was estimated to be 0.139 (95% CI, 0.127 to 0.151) and the *R* was estimated as 0.555 (95% CI, 0.509 to 0.602) (Table 1 and Figure 3).

### COVID-19 model for Daegu and North Gyeongsang Province

**Figure 4.**
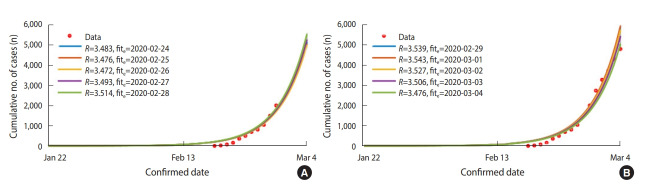
Estimated reproductive number (*R*) by daily cumulative confirmed patients in Daegu and North Gyeongsang Province from February 18, 2020 to March 4, 2020. The first patient was assumed to have been infected on January 22, 2020. The fit_e_ refers to the last date of the model fitting. Cumulative number of cases (red dots) and model fitting curves (colored lines). (A) fit_e_: February 24, 2020 to February 28, 2020 (B) fit_e_: February 29, 2020 to March 4, 2020

Daegu and NGP were showing a pattern of rapid increase since the first case was confirmed on February 18, 2020. Under the assumption that it began with one infected person expressing symptoms on January 22, 2020, the *R* was estimated with data fitting using the number of daily confirmed cases in Daegu and NGP from February 18, 2020 to March 4, 2020. Depending on the fitting period, the *R* starts at 3.483 (based on the cases confirmed from February 18, 2020 to 24, 2020), increases to 3.543 (based on the cases confirmed from February 18 to March 1, 2020) and then decreases to 3.476 (based on the cases confirmed from February 18 to March 4, 2020) (Figure 4).

The effects of reduced *β* and transmission period (period from symptom onset to confirmation, 1/*α*) due to preventive measures were analyzed. These are the parameters of *R* calculated above; in order for *R* to be less than 1, the *β* must be reduced by at least 72% or the transmission period must be below 28 hours (Appendix 1). Taking these into account, the scenario for the effect of preventive measures assumed a 75%, 90%, and 99% reduction in *β*, and the transmission period was reduced from 4 days to 2 days. In addition, a comparison was made by assuming that the effect of these preventive measures showed from March 5, 2020 or February 29, 2020. The data were simulated based on the daily confirmed cases from February 18 to 28, 2020, and some of the scenarios were selected and presented in Table 2. The results of simulating the scenario on the effects of preventive measures assumed that an additional large-scale group infection in Daegu and NGP would not occur.

Under the assumption that the effects of preventive measures appear from March 5, 2020, reducing the *β* by 90% or 99% brought the peak of the epidemic forward to March 7, 2020 and March 6, 2020, with the total number of patients reduced to 26,634 and 19,426, respectively. When the *β* was reduced by 99% and the transmission period was also reduced to 2 days, the peak of the epidemic occurred a day earlier on March 5, 2020 but the number of daily confirmed cases at the peak of the epidemic increased to 2,425 and the total number of patients slightly decreased to 19,403. However, the point at which daily confirmed cases is equal to or below 10 was brought forward from April 5, 2020 to March 30, 2020, essentially shortening the overall duration of the epidemic.

**Figure 5.**
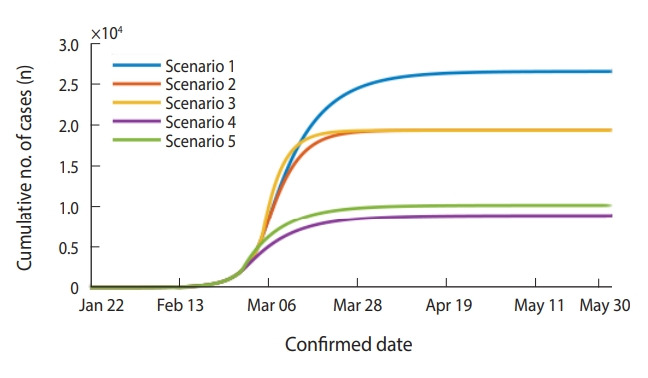
Estimated number of cumulative confirmed patients by scenario in Daegu and North Gyeongsang Province (see Table 2 for scenarios).

Assuming that the effects of the preventive measures appear from February 29, 2020, when the transmission period was reduced to 4 days and the *β* was reduced by 90%, the peak of the epidemic occurred earlier on March 2, 2020 and the total number of patients was also reduced to 8,894. However, the pattern of incidence until March 4, 2020 makes most sense when the transmission period is 2 days and the *β* is reduced by 75% with the assumption that the effects of preventive measures appear from February 29, 2020. At this point, the peak of the epidemic was February 29, 2020 with the number of daily confirmed cases reaching 819 and the total number of patients estimated to be approximately 10,249. The point in time at which daily confirmed cases drop below 10 cases per day was estimated as April 10, 2020. In other words, the total duration of an epidemic is shortened when the transmission period is reduced from 4 days to 2 days, but this increases the size of the peak of the epidemic. However, there was no significant difference in the total number of patients. It was found that the reduction in *β* also reduced the total duration of the epidemic and the size of the peak of the epidemic, thereby reducing the total number of patients (Table 2 and Figure 5). The pattern of change in the number of patients according to the date of confirmation for each scenario was presented in the appendix along with the basic scenario (Appendices 2 and 3).

## DISCUSSION

The world is undergoing a COVID-19 epidemic. Although the WHO is delaying official declaration, the signs indicating that a pandemic has already begun are showing up not only in Asia, but also in Europe, the Middle East, Africa, and South America. The *R* is an important epidemiologic factor in evaluating the level of epidemics and selecting appropriate prevention and intervention policies. However, in the case of COVID-19, various pieces of epidemiological information including *R* is not known as it is a new infectious disease, and it is necessary for each country to quickly review them as they change according to the progress of the epidemic. In Korea, the number of infected people continuously increases every day since the first case on January 20, 2020. The results of the study indicate that the values of *R* for COVID-19 are significantly different at the early stage without spreading within the community (from January 19, 2020 to February 10, 2020, based on symptom onset date) and in the case of Daegu and NGP where the number of confirmed cases are rapidly increasing since the diagnosis of patient #31 on February 18, 2020.

The initial *R* for Korea was about 0.56, which was significantly less than the *R*=3.6 minus 4.6 calculated for the epidemic in Hubei, China, and also markedly smaller than the estimates that have previously been published (from 2.2 [95% CI, 1.4 to 3.9] to 3.58 [95% CI, 2.89 to 4.39]) [5,6]. Even considering the errors arising from the undetected cases in the initial stage of the epidemic, the initial estimate of *R* for Korea shows that the infection was effectively controlled as a result of the intervention policy such as the screening by the quarantine authorities, patient isolation, and management of whose who came in contact with the infected.

After February 18, 2020, the *R* for the Daegu/NGP epidemic was 3.5, which was less than the value during the initial epidemic in Hubei, China (approximately *R*=4) but was still far greater than the national value in the initial stage. The case of Daegu/NGP shows the spread of COVID-19 under special circumstances. It is postulated that the number of people infected with COVID-19 increased sharply after people attended the massive religious service at Shincheonji Church in Daegu/NGP. In addition, the case of transmission within the Cheongdo Daenam Hospital also shows *R* that is significantly higher than that of the national estimate due to the continued close contact between patients and medical staff in a confined space. The number is much higher than 2.28 (95% CI, 2.06 to 2.52), which was the *R* for the initial stage in a study on the Japanese cruise ship (Diamond Princess) [7]. This study shows the *R* for the transmission between humans without transmission by animals compared to previous studies [5,6] in an environment that perfectly matches the premise for an infectious disease model in which the total population is relatively fixed. For the case of Daegu/NGP, it can be seen that the β was greater than that within the cruise ship due to the close contact that occurred under special circumstances.

The results of predicting *R* for Daegu/NGP suggested that only using preventive measures such as keeping track of those who came in contact with the infected, as used in the early stage of COVID-19 transmission, was insufficient to prevent the spread in the region. As demonstrated by the results of the simulation, it is possible to reduce the overall size of the epidemic as well as the number of patients during the peak period with a strict prevention policy that reduces COVID-19 transmission, in addition to the initial prevention method. To achieve this, it is necessary to implement the preventive measures that reduce the probability of transmission upon contact with an infected person, the level of contact, and the duration of transmission, all of which affect the *R*. For example, in order to reduce the probability of transmission upon contact with an infected person, it is necessary to wear a mask when visiting a crowded place or a medical institution, and to engage in social distancing to limit contact with people as much as possible. Also, the current prevention policy of quickly identifying, diagnosing, and isolating infected people is essential to reduce the transmission period. As there is no treatment for COVID-19 with verified effects yet, the transmission period cannot be reduced by using treatment for patients, as in the case for influenza.

The modeling results in this study do not take into account those who are under self-quarantine upon contact with an infected person or the transmission by an asymptomatic patient, and do not show the effects of transmission by and management of asymptomatic infectious people, or the effects of managing those under self-quarantine. As shown by Tang et al. [8], management of asymptomatic infectious people through intensive tracking of those who have been exposed and self-isolation are both effective in reducing the *R*. The quarantine authorities have upgraded the infectious disease crisis warning to the highest level (‘Serious’) on February 23, 2020, and are implementing special preventive measures in Daegu/NGP. In addition to preparing a dedicated team for managing self-isolators to ensure they remain in isolation, preventive policies such as securing the list of names for Shincheonji Daegu Church attendees to test and isolate them and banning Shincheonji gatherings, which were the source of massive transmission, are being implemented with the expectation to show results soon.

The limitations of this study are as follows: first, the symptom onset dates for patients could not be used in the Daegu/NGP model and only the dates for confirmed diagnosis were used. Therefore, there is a possibility that the predicted schedule for actual patient occurrence may appear earlier than in the results of this study. Second, there may be errors due to fixing the period from symptom onset to diagnosis to 4 days and 2 days in the analysis. This is because the period between symptom onset and diagnosis showed a tendency to become shorter as the epidemic progressed, following the government’s active preventive measures and the increased awareness of symptoms among the infected with a rapid increase in COVID-19 patients. However, due to individual differences, it could not be adjusted uniformly the period from symptom onset to diagnosis. In the future, a model that supplements the data on the symptoms and diagnosis dates for each individual is needed. Third, to simplify mathematical modeling, transmission during latency or asymptomatic infections were not taken into account, and it was assumed that the transmission of infection does not occur when patients are isolated. In addition, this model assumed that there was no inflow of infections from abroad, so it is possible that the number of infected patients is estimated to be smaller than it actually is, and there is a possibility that the epidemic will continue depending on the influx of infected cases from abroad. For the case of Daegu/NGP, assumption was also made that a massive group infection would not occur additionally in the future and the actual pattern of the epidemic may be different from the results of the model if a future largescale group infection occurs. Lastly, the model only includes Daegu/NGP and not the entire population in Korea. This study focused on Daegu/NGP as this region accounts for 90% of the current epidemic, but the pattern of the epidemic might be very different from those of other regions (Appendix 4). Thus, in the future, a detailed regional modelling based on the entire population of Korea should be added.

In conclusion, the early stage epidemic in Korea was controlled at *R*=0.5 for the initial 30 days with approximately 30 patients but the epidemic in Daegu/NGP starting with patient #31 appeared rapidly in large-scale with *R*=3.5. However, the transmission period is becoming shorter with active COVID-19 testing by the quarantine authorities, and *β* is decreasing with active participation of the citizens in preventive measures such as wearing a mask and social distancing. In order to resume normal life by reducing the size of the epidemic and reducing the transmission period to minimize the number of patients, active participation in preventive activities on the part of the citizens is required in addition to continued efforts by the quarantine authorities. In particular, it is necessary to strictly control transmission in group facilities where vulnerable individuals are gathered to prevent local outbreaks. In addition, since the epidemic has spread throughout the world except for China, continued efforts are necessary to prevent the influx of new cases from abroad. Our research team for infectious disease modeling will continue to accumulate new information to modify and develop modeling research results.

## Figures and Tables

**Table 1.** Parameters of the coronavirus disease 2019 (COVID-19) transmission model in Korea

Symbol	Description	Value	Reference
*β*	Transmission rate	0.139^1^	Data fitted
*κ*	Progression rate	1/4	[4]
*α*	Isolation rate	1/4	[4]
*γ*	Removal rate for isolated individuals	1/14	[4]

1 The β has been estimated from the early stage COVID-19 epidemic model in Korea from the Results section.

**Table 2.** Estimated changes in the peak and size of coronavirus disease 2019 epidemic according to the effect of preventive measures using mathematical modeling in Daegu and North Gyeongsang Province, 2020

Scenario	Preventive measures			Peak day	Confirmed case at peak day (n)	Less than 10 confirmed case per day	Less than 1 confirmed case per day	Total confirmed case (n)^1^
	Effect start date	Transmission duration (d)	Transmission rate reduction (%)					
Base	None	4	0	Apr 5	22,389	Jun 14	Jun 28	4,992,000
1	Mar 5	4	90	Mar 7	1,454	Apr 27	May 20	26,634
2	Mar 5	4	99	Mar 6	1,390	Apr 5	Apr 16	19,426
3	Mar 5	2	99	Mar 5	2,425	Mar 30	Apr 8	19,403
4	Feb 29	4	90	Mar 2	485	Apr 12	May 4	8,894
5	Feb 29	2	75	Feb 29	819	Apr 10	May 1	10,249

1 Cumulative number of confirmed patients to less than one confirmed case per day.

## SUPPLEMENTARY MATERIALS

Korean version is available at http://www.e-epih.org/.

## Appendix 1.

A contour map of the reproductive number (*R*) as transmission rate and transmission duration changes. *R*=3.5 (red spot) and *R*=1 (black line).

## Appendix 2.

Estimated reproductive number (*R*) and daily number of confirmed patients in Daegu and North Gyeongsang Province at base scenario which means no preventive measures. *R* varies with fitting period. Number of cases (red dots) and model fitting curves (colored lines).

## Appendix 3.

Estimated daily number of confirmed patients by scenario in Daegu and North Gyeongsang Province. See Table 2 for scenarios. Number of cases (red dots) and model fitting curves (colored lines).

## Appendix 4.

Cumulative confirmed number of patients and ratio by region on March 8, 2020. (A) and (B) the national distribution of the cumulative confirmed cases in Korea; (C) the ratio of cumulative confirmed cases by region; Gyeongbuk is North Gyeongsang Province.
