# Supplementary material for: Estimating the reproductive number and the outbreak size of COVID-19 in Korea
Source: Epidemiol Health. 2020 Mar 12;42:e2020011. doi: 10.4178/epih.e2020011 (PMC7285447; doi:10.4178/epih.e2020011)
Supplement: Supplementary file 1 [file epih-42-e2020011-suppl.docx]

**Estimating the reproductive number and the outbreak size of novel coronavirus disease 2019(COVID-19) in Korea**

(한국 코로나-19 감염질환 유행 자료를 이용한 감염재생산수와 유행 규모 추정)

Sunhwa Choi^1^, Moran Ki^1^

^1^Department of Cancer Control and Population Health, Graduate School of Cancer Science and Policy, National Cancer Center, Goyang, Korea

최선화, 기모란

국제암대학원대학교 암관리학과, 국립암센터, 고양, 한국

**Correspondence to:** Moran Ki, moranki@ncc.re.kr

ORCID

Sunhwa Choi, http://orcid.org/[0000-0002-6608-6981](http://orcid.org/0000-0002-6608-6981)

Moran Ki, http://orcid.org/0000-0002-8892-7104

한글초록:

목적(Objectives): 한국에서 코로나-19 첫환자가 2020년 1월 20일 진단된 이래 2월 17일까지 약 1달간 30명의 환자가 진단되었다. 그런데 2월 18일 31번째 환자가 확진된 이후 3월4일까지 15일간 5,298명이 추가로 확진 되었다. 이에 수학적 모델링에 의한 감염재생산수(reproductive number, $\mathcal{R}$)를 추정하고 전체 유행의 규모와 유행 종료 시점 등 코로나-19 확산 양상을 예측하고자 한다.

방법(Methods): 결정론적 수학적 모델(SEIHR)에 기반한 코로나-19확산 모델을 한국 유행 상황에 맞게 구축하였다. 한국의 확진자 90%가 대구와 경북에서 발생하였기에 이 지역을 기준으로 일별 확진자수를 사용하여 유행확산을 예측하였다. 다만 대구 경북 유행의 첫 환자 발병일은 1월 22일로 가정하였다. 방역조치가 없었던 경우에 비하여 방역조치를 통하여 감염전파율과 감염전파기간(증상발생부터 확진(격리)까지의 기간)을 줄였을 경우, 방역조치 효과 시작일에 따른 $\mathcal{R}$ 과 유행 규모를 추정하였다.

결과(Results): 중국 후베이성에서의 코로나-19의 $\mathcal{R}$ 은 4.0281, 한국에서 추정된 초기 감염재생산수는 0.5555 였으나 이후 대구/경북의 경우는3.4721 - 3.5428사이 값을 가진다. 방역조치로 인하여 감염 전파기간이 4일에서 2일로 감소되면 총 유행기간은 감소하지만, 유행정점의 크기는 커지고 총 환자수 규모는 큰 차이가 없었다. 전파율이 감소되면 총 유행기간도 감소하고 유행 정점의 크기도 감소하며, 총 환자수도 감소하는 것으로 나타났다.

결론(Conclusions): 코로나-19 유행의 빠른 종식을 위해서는 방역당국의 노력으로 가능한 빠르게 환자를 찾아서 격리하여 감염전파기간을 줄이는 정책과 함께 국민 참여로 사회적 거리두기, 마스크 쓰기 등의 전파율 감소 노력이 절대적으로 중요하다. 수학적 모델링에 의한 $\mathcal{R}$ 추정과 확산예측은 코로나-19전파 가능성과 심각성에 따른 질병 중재 정책의 유형과 강도를 결정하는데 중요한 정보를 제공할 수 있다.

중심단어: 코로나-19(COVID-19), 역학, 수학적 모델, 유행, 감염재생산수, 대한민국

**서론(Introduction)**

2019년 12월 말 중국에서는 원인불명 폐렴 환자가 발생하였다고 보고하였고, 이후 이 질환은 신종코로나바이러스에 의한 것으로 밝혀졌으며 WHO는 새로운 이 질환을 COVID-19라 명명하였다. 2020년 3월 8일 현재 전세계적으로 10만명 정도 감염자가 발생하였고, 한국에서도 7000건이 넘는 감염자가 확진 되었다. [1, 2]

한국은 2020년 1월20일 첫 감염자 발생을 시작으로 2월17일까지 약 30일간 30명의 감염자가 확진 되었고, 이 중의 대부분이 해외에서 감염되어서 국내로 들어왔거나, 이들 지표환자로부터 국내에서 감염된 사례들이다. 그런데 2020년 2월18일 31번째 환자를 시작으로 대구, 경북지역을 중심으로 전례없이 빠르고 큰 규모의 유행 양상이 나타나고 있다: 2월18일 31번 환자 발생후 접촉자 추적하는 과정에서 이 환자가 신천지 대구교회를 방문한 것으로 확인되었고, 2월9일과 16일 신천지 대구예배에 참석했다는 사실이 확인되면서 신도들에게 확산되었을 가능성이 제기됐다. 2월 20일 신천지 대구교인 1천여명에 대해 전수 조사에 들어갔다. 또한, 31번 확진자가 2월 초 경북 청도에 방문한 것을 확인하고 청도 대남병원과의 연관 가능성도 조사하기 시작하였다. 이후, 대구/경북을 중심으로 코로나-19 확진환자가 급증하기 시작하여 31번째 환자가 확진된 이후 3월4일까지 15일간 5,298명이 추가로 확진 되었다. 방역당국은 2월 23일 감염병 위기경보를 심각 단계로 상향하여 적극 대응을 취하고 있다. 따라서 초기의 한국에서의 코로나-19감염재생산수와 대구, 경북의 감염재생산수 추정을 통해 유행 규모를 추정하고 현재 및 미래에 필요한 중재정책을 결정하는데 중요한 정보를 제공할 필요가 있다.

방법(Methods)

1. 자료 (data)

수학적 모델의 모수 추정을 위하여 질병관리본부의 보도자료를 분석하여 2020년 1월20일부터 3월 4일까지의 일별 확진자 데이터를 사용하였다 [2]. 한국의 총 인구, 대구, 경북의 인구 데이터는 국가통계포털의 2019년 인구수를 이용하였다 [3]. 중국의 일별 확진자수는 중국 정부의 공식 보고자료를 활용하였다.

1. 수학적 모델 (mathematical model)

코로나-19 감염전파 모델은 결정론적 모델로 SEIHR 모델을 기반으로 구성된다. 이 모델에서 인구 그룹은 질병에 걸릴 수 있는 감수성자 그룹(S), 잠복 감염자 그룹(E), 증상이 있는 감염자 그룹(I), 확진 되어 격리된 그룹(H), 회복자 그룹(R) 등 5개의 그룹으로 나누어진다. (Fig. 1)


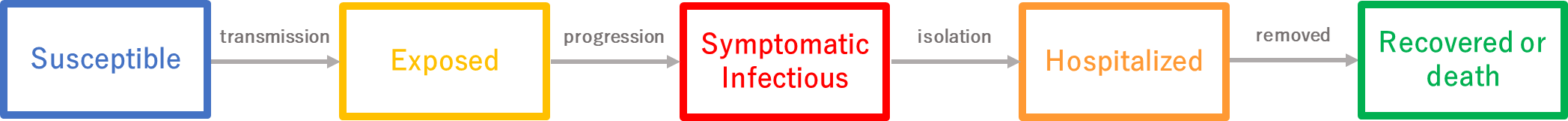


**Fig. 1. 코로나-19 전파 결정론적 SEIHR모델, 감수성자 그룹(S), 잠복 감염자 그룹(E), 증상이 있는 감염자 그룹(I), 확진 되어 격리된 그룹(H), 회복자(또는 사망) 그룹(R).**

이 모델의 가정은 다음과 같다.

1. 인구의 출생과 자연 사망은 고려하지 않음
2. 잠복기 감염과 무증상 감염 전파는 고려하지 않음
3. 확진 후 격리되어서 치료하는 동안은 질병을 전파시키지 않음
4. 회복된 사람은 재감염 되지 않음

감수성자 그룹은 증상이 있는 감염자 그룹과의 접촉을 통해 감염되어 잠복 감염자 그룹으로 이동된다. 코로나-19의 감염 전파수학적 모델은 다음과 같이 표현된다. 전체 인구(N)는 각 그룹별 인구의 합과 같다. 여기서 각 그룹별 인구의 시간에 따른 변화는 아래 수식1과 같다. (Equation 1)

모델 상수 $\beta$는 감염 전파율을 의미하며, 잠복 감염자는 일정기간이 지난후에 증상이 발현되어 전파율이 있는 감염환자로 발전하게 된다. 상수 $\kappa$는 코로나-19의 증상발현 진행률을 의미하고 $1/\kappa$은 코로나-19의 평균 잠복기간을 나타낸다. 증상이 발현된 감염자는 일정기간 이후에 확진 되어 격리된다. 증상 감염자의 증상발현 후 확진되어 격리되는 속도는 $\alpha$로 표기하였으며 $1/\alpha$는 증상 발현 이후 확진 되기까지의 평균 기간이다. 이는 곧 감염 전파 가능 기간이 된다. 상수 $\gamma$는 격리 감염자의 회복률을 나타내며 $1/\gamma$는 회복되기까지의 평균 격리기간을 의미한다.

$$\frac{dS}{dt}=-\beta\frac{I}{N}S,$$

$$\frac{dE}{dt}=\beta\frac{I}{N}S-\kappa E,$$

$$\frac{dI}{dt}=\kappa E-\alpha I,$$

$$\frac{dH}{dt}=\alpha I-\gamma H$$

$$\frac{dR}{dt}=\gamma H,$$

$$N=S+E+I+H+R.$$

Equation 1. 코로나-19 유행 예측 모델에서 SEIHR그룹별 인구의 시간에 따른 변화를 보여주는 미분방정식.

코로나-19 감염 전파 모델의 모수 정의와 값은 Table 1과 같다. 증상발현 진행률($\kappa$) , 격리율($\alpha$), 회복율($\gamma$) 은 각각 정의에 따라 잠복감염기간(4일), 증상 발현 이후 확진 되기까지의 평균 기간(4일), 회복되기까지의 평균 격리기간(14일)을 우리나라의 초기 확진자 데이터 분석 결과 값을 참고하였다 [4].

감염전파율($\beta$)는 일별 누적 확진자수 데이터와 모델에서의 해당 날짜의 누적 확진자 수를 최소제곱법(least square fitting method)을 사용하여 데이터($x$) 와 모델에서 추정되는 확진자 수($\alpha I$) 차이의 제곱을 최소화하는 감염전파율을 추정하였다. 즉, $\sum_{i} {(cumulative(x)-{cumulative(\alpha I))}_{i})}^{2}$가 최소가 되는 감염전파율을 Matlab의 “lsqcurvefit” 패키지를 사용하여 추정하였다. (Table 1)

**Table 1. Parameters of the novel coronavirus disease (COVID-19) transmission model in South Korea**

| Symbol | Description | Value | Reference |
| --- | --- | --- | --- |
| $\beta$ | 감염전파율 (Transmission rate ) | 0.1389* | data fitted |
| $\kappa$ | 증상발현 진행률(Progression rate ) | 1/4 | [4] |
| $\alpha$ | (격리율) Isolation rate | 1/4 | [4] |
| $\gamma$ | (회복율) Removal rate for isolated individuals | 1/14 | [4] |

* 감염전파율은 결과(Results)의 한국의 코로나-19 초기 유행 단계 모델에서 추정된 값

1. 감염재생산수 (Reproduction number)

감염재생산수($\mathcal{R}$)란 감염된 환자 1명이 감염전파기간동안 감염시키는 사람 수를 의미한다. $\mathcal{R}$ 이 1보다 크면 감염 유행이 커지고, $\mathcal{R}$ 이 1보다 작으면 유행이 감소하는 시기임을 알 수 있다. $\mathcal{R}$ =1인 경우는 지역사회에서 일정 수준의 환자가 지속적으로 발생하는 감염질환의 토착화(endemic)가 되었음을 의미한다.

$\mathcal{R}$ 은 감염자와 접촉시 감염이 전파될 확률(probability), 감염자와의 접촉 횟수(contacts), 감염자가 감염을 전파시킬 수 있는 기간(duration)에 의해 결정된다. 이때 감염이 전파될 확률과 접촉 수준을 곱해서 전파율로 설명하기도 한다. 이 연구의 코로나-19 감염 수학적 모델에서 계산한 코로나-19의 감염재생산수($\mathcal{R}$)는 다음과 같다. 이때 $\beta$는 전파율이고 $\frac{1}{\alpha}$ 은 감염전파기간을 의미한다.

$$\mathcal{R}= \frac{\beta}{\alpha}$$

중국 후베이성 코로나-19 유행 초기단계의 $\mathcal{R}$ 을 추정하기 위하여 2019 년 12 월 29 일부터 2020 년 2 월 3 일까지의 일별 확진 데이터를 사용하였다.

한국 코로나-19 유행 초기 단계의 $\mathcal{R}$ 을 추정하기 위하여는 2020 년 1 월 20 일부터 2020 년 2 월 17일 까지의 30명 확진자의 증상 발현일 자료를 사용하였다. 또한, 한국에서 감염된 경우와 해외에서 감염되어 한국으로 들어온 경우를 구분하여 해외유입함수를 모델에 적용하였다. 해외유입 감염자의 경우 증상 발현일에 코로나-19 전파모델(fig 1 참조)에서 그룹 $I$ 로 들어온 것으로 적용하였다. 해외유입 감염자가 증상 발현일 이후 한국에 입국한 경우에는 입국일에 그룹 $I$ 로 들어온 것으로 적용하였다.

한국의 대구/경북 지역에서는 중국 우한을 봉쇄한 1월 23일 전날인 1월22일 증상발현 감염자 1명이 들어온 것으로 가정하였고, 2020 년 2 월 18 일부터 3 월 4일까지 대구와 경북의 일별 확진자 데이터를 사용하여 $\mathcal{R}$ 을 추정하였다. 이때 시간에 따라 $\mathcal{R}$ 이 변하므로 데이터 피팅 기간을2월18일부터 2월24일을 기준으로 계속 하루씩 늘려가면서 새롭게 계산하여 $\mathcal{R}$ 의 변화를 검토하였다.

**결과(Results)**

1. 중국 후베이성에서의 코로나-19 초기 유행 단계 모델

후베이성의 전파율은0.8056 (95% CI, 0.8020 - 0.8093)이고, 감염재생산수는 4.0281 (95% CI, 4.0100 - 4.0465)로 추정되었다.

또한 후베이성의 유행 시작 시점이 불분명 하므로 12월 29일에 1명이 아니라 1명에서 5명까지 감염환자(I0)가 있었을 것으로 가정하면 아래 그림과 같이 $\mathcal{R}$ =3.6387 (초기환자 5명인 경우)부터 $\mathcal{R}$ =4.6084 (초기환자가 1명인 경우)로 변화한다. (Fig.2)

**Fig. 2.** **중국 후베이성의 코로나-19 일별 누적 확진자 수 (붉은색 점)에 따른 감염재생산수(**$\mathcal{R}$**) 모델 피팅 곡선(실선). 12월 29일 초기 감염자수는 1명에서 5명으로 가정함.**

1. 한국의 코로나-19 초기 유행 단계 모델

한국의 초기 환자 30명 발생까지를 모델링 한 결과 코로나-19 전파율은 0.1389 (95% CI, 0.1272 - 0.1506)이고, 감염재생산수는 0.5555 (95% CI, 0.5086 - 0.6024)로 추정되었다. (Fig.3, Table 1)

**Fig. 3.** **한국 2020년1월20일-2월17일까지 증상 발현일에 따른 코로나-19 누적 확진자 수 (붉은색 점)와 감염재생산수(**$\mathcal{R}$**)계산을 위한 모델 피팅 곡선(실선)**

1. 대구와 경북의 코로나-19 유행 모델

대구와 경북 지역에서는 2020년 2월 18일 첫 감염자가 확진 된 이후, 급격히 증가하는 양상을 보이고 있다. 1월22일 증상발현 감염자 1명으로 시작되었다고 가정하고, 2020 년 2 월 18 일부터 3 월 4일까지의 대구/경북에서의 일별 확진자수 자료를 사용하여 데이터 피팅을 통하여 감염재생산수를 추정하였다. 피팅 기간에 따라 감염재생산수는 $\mathcal{R}$ =3.4831로 시작 (2월18일부터 2월24일 확진자 기준)하여, $\mathcal{R}$ =3.5428 (2월18부터 3월1일 확진자 기준)까지 증가하다 $\mathcal{R}$ =3.4764 (2월18부터 3월4일 확진자 기준)로 감소하는 경향을 보인다.(Fig. 4)

|  |
| --- |
|  |

**Fig. 4. 대구와 경북 지역의 2020년2월18일부터 3월4일까지 일별 코로나-19 누적확진자 수(붉은색 점)와 감염재생산수(**$\mathcal{R}$**) 계산을 위한 모델 피팅 곡선(실선). 첫 환자는 1월 22일에 감염된 것으로 가정하였고 fit_e_는 모델 피팅 마지막 날짜를 의미함.**

방역조치로 인하여 전파율($\beta$)과 감염전파기간(증상 발현일부터 확진일까지의 기간, $1/\alpha$ )이 감소하였을 때의 효과에 대해 분석하였다. 이는 앞에서 계산된 $\mathcal{R}$ 를 구성하는 모수로, $\mathcal{R}$이 1보다 작아지려면 전파율을 72%이상 감소시키거나, 감염전파기간은 28시간이하로 줄여야 한다(App_Fig. 1). 이러한 상황을 고려하여 방역조치로 인한 효과의 시나리오는 전파율이 75%, 90%, 99% 감소되었을 때를 가정하였고, 감염전파기간이 4일과 2일로 감소되는 것을 가정하였다. 또한 이러한 방역조치 효과가 3월 5일부터 나타나는 것과 2월 29일부터 나타나는 것을 가정하여 비교하였다. 자료는 2월18일부터 2월28일까지의 일별 확진자 기준으로 시뮬레이션 하였다. 이중 몇 가지 시나리오를 선별하여 표2에 제시하였다. 방역조치 효과에 대한 시나리오 시뮬레이션의 결과는 추가적인 대구/경북에서 대규모 집단감염이 발생하지 않음을 가정하였다.

방역조치 효과가 3월 5일부터 나타난다는 가정하여 전파율이 90% 또는99% 감소시에는 유행의 정점이 3월 7일과 3월 6일로 각각 앞당겨 지고 총 환자수는 26,634명, 19,426명으로 각각 감소되는 것으로 나타났다. 전파율이 99% 감소되면서 전파기간도 2일로 줄어들면 유행의 정점은 3월 5일로 하루 앞당겨지지만 유행 정점시 하루 확진자수가 2,425명으로 많아지고 총 환자수는 19,403명으로 조금 감소하였다. 하지만 하루 확진자 10명 이하시점은 4월 5일에서 3월 30일로 앞당겨져 전체 유행기간이 줄어드는 효과가 나타났다.

방역조치 효과가 2월 29일부터 나타난다는 가정을 하고, 전파기간은 4일, 전파율은 90% 감소되었을 때는 유행의 정점은 3월 2일로 앞당겨 지고 총환자수도 8,894명으로 감소되었다. 그런데 3월 4일까지의 환자발생수 양상과 가장 잘 맞는 것은 방역조치 효과가 2월 29일부터 나타난다는 가정과 함께, 전파기간은 2일, 전파율은 75% 감소되었을 때로 이때 유행의 정점은 2월 29일로 하루 확진자가 819명이고 총 환자수는 약 10,249명으로 추정되었다. 하루 확진자가 10명이하로 떨어지는 시점은 4월 10일로 추정되었다. 즉, 감염 전파기간이 4일에서 2일로 감소되면 총 유행기간은 감소하지만, 유행정점의 크기는 커지는 것으로 나타났다. 그러나 총 환자수 규모는 큰 차이가 없었다. 전파율이 감소되면 총 유행기간도 감소하고 유행 정점의 크기도 감소하며, 총 환자수도 감소하는 것으로 나타났다. (Table 2, Fig.5) 기본 시나리오와 함께 시나리오별 확진일에 따른 환자수 변화양상은 Appendix에 그림으로 제시하였다. (App_Fig. 2, App_Fig.3)

**Table 2.** **대구와 경북 코로나-19유행에서 방역조치 효과에 따른 유행 정점과 확진자 발생수준 변화 양상**

| 시나리오 | 방역조치 효과 | | | 유행정점 | 유행정점시  하루 확진자 수 | 하루 확진자  10명이하 시점 | 하루 확진자 1명이하 시점 | 총 환자수* |
| --- | --- | --- | --- | --- | --- | --- | --- | --- |
|  | 효과시점 | 전파기간 $1/\alpha$ | 전파율  $\beta$  감소 |  |  |  |  |  |
| 기본 | 없음 | 4일 | 0 | 4월5일 | 22,389명 | 2020-06-14 | 2020-06-28 | 4,992,000 |
| 1 | 3월5일 | 4일 | 90% | 3월7일 | 1,454명 | 2020-04-27 | 2020-05-20 | 26,634 |
| 2 | 3월5일 | 4일 | 99% | 3월6일 | 1,390명 | 2020-04-05 | 2020-04-16 | 19,426 |
| 3 | 3월5일 | 2일 | 99% | 3월5일 | 2,425명 | 2020-03-30 | 2020-04-08 | 19,403 |
| 4 | 2월29일 | 4일 | 90% | 3월2일 | 485명 | 2020-04-12 | 2020-05-04 | 8,894 |
| 5 | 2월29일 | 2일 | 75% | 2월29일 | 819명 | 2020-04-10 | 2020-05-01 | 10,249 |

*하루 확진 1명이하 시점까지의 누적 확진자 수

******

**Fig. 5.** **대구와 경북 지역의 시나리오 별 코로나-19 누적 확진자 수. 시나리오는 Table 2 참조**

**토론(Discussion)**

전세계가 코로나-19 유행을 겪고 있다. WHO가 공식 선언을 미루고 있지만 이미 팬데믹(pandemic)이 시작되었다고 할 수 있는 징후는 아시아 뿐 아니라 유럽, 중동의 유행과 아프리카, 남아메리카 유행에서도 나타나고 있다. 감염재생산수($\mathcal{R}$)는 유행의 수준을 평가하여 적절한 방역 및 중재정책을 선택하는 데 중요한 역학 변수이다. 그러나 코로나-19의 경우 신종감염병으로 $\mathcal{R}$ 을 비롯한 여러가지 역학적 정보가 알려져 있지 않고, 유행 상황에 따라 달라지므로 국가별로 이에 대한 빠른 검토가 필요한 상황이다. 국내에서는 2020년 1월 20일 첫 감염자 발생을 시작으로 현재까지 코로나-19 감염자는 계속 증가하고 있다. 연구 결과 국내에서의 코로나-19의 $\mathcal{R}$ 은 지역사회확산이 일어나지 않았던 초기(증상 발현일 기준 1월19일부터 2월 10일)와 2월18일 31번째 확진자 진단 후 감염자가 급격히 증가하고 있는 대구/경북의 경우는 확연히 다른 값을 가진 것으로 나타났다.

국내에서의 초기 $\mathcal{R}$ 은 0.56정도로 그 당시 중국의 후베이성 유행에서 계산된 $\mathcal{R}$ =3.6~4.6보다 훨씬 작았고, 이는 이전에 발표된 추정치 2.2 (95 % CI, 1.4–3.9)에서 3.58 (95 % CI, 2.89–4.39) 보다도 훨씬 작다[5,6]. 이는 질병 발생의 초기 단계에서 다수의 감염된 사례가 확인되지 않았기 때문에 발생하는 오차를 감안하더라도, 국내에서의 초기 $\mathcal{R}$ 추정치는 방역당국의 검역, 환자격리, 접촉자 관리 등과 같은 중재정책이 포함되어 있는 결과로 감염관리가 잘 되었음을 보여준다.

2월 18일 이후 대구/경북 유행에서는 $\mathcal{R}$ =3.5정도로 중국의 후베이성 초기 유행에서의 $\mathcal{R}$ 인 4보다는 작지만, 초기의 우리나라의 초기 유행시 $\mathcal{R}$ 값보다 훨씬 큰 값을 보였다. 대구/경북 경우는 특수한 상황에서의 코로나-19의 감염확산을 보여준다. 대구/경북에서는 신천지 종교 집회에 참석하여 감염자들이 신도들과의 밀접접촉을 통해 코로나-19감염자가 급격히 증가한 것으로 추정된다. 또한, 경북 청도대남병원에서의 병원내 감염전파 사례 역시 한정된 공간에서 지속적으로 밀접접촉이 발생하여 환자와 의료진 대부분이 감염되어 초기의 우리나라의 $\mathcal{R}$ 보다 훨씬 높은 값을 나타낸 것으로 보인다. 이는 일본 크루즈(다이아몬드 프린세스호) 내에서의 초기 단계 $\mathcal{R}$ 값을 분석한 연구에서 나온

결과인 2.28 (95 % CI, 2.06–2.52) 보다 큰 값을 나타낸다[7]. 이 연구는 감염병 모델의 전제와 완벽하게 일치하는 총 탑승 인원수가 상대적으로 고정되어 있고, 선행 연구[5,6]와 비교하여 동물감염이 없는 사람간 전파에 의한 $\mathcal{R}$ 값을 나타낸다. 대구/경북의 경우는 특수한 상황에서의 밀접접촉으로 인해 감염 전파율이 일본 크루즈내에서의 감염전파율보다 컸음을 알 수 있다.

대구/경북에서 $\mathcal{R}$ 예측 결과는 대구/경북에서의 방역은 국내 코로나-19의 확산 초기단계의 방역과 같이 접촉자 관리만을 통해서는 확산을 방지하는데 어려움이 있음을 알 수 있었다. 시뮬레이션 결과에서 보듯이 초기 방역 방식과 더불어 코로나-19 감염전파율을 줄이는 강력한 방역정책을 통해 전체 유행의 규모를 줄이고 정점시기의 환자수를 줄일 수 있다. 이를 위하여 $\mathcal{R}$ 에 영향을 미치는 감염자와 접촉시 감염확률(probability), 접촉수준(contacts), 감염전파기간(duration)을 모두 감소시킬 수 있는 방역 조치가 필요하다. 예를 들어 감염자와의 접촉시 감염확률을 줄이기 위하여 사람들이 밀집된 환경이나 의료기관 방문시 마스크를 사용하는 것과 접촉 수준을 줄이기 위하여 가능한 사람들과의 접촉을 줄이는 사회적 거리두기(social distancing)가 필요하다. 또한 감염자의 감염전파기간을 줄이기 위하여 감염자를 최대한 빠르게 찾아내어 진단하고 격리하는 현재의 방역 정책도 필요하다. 코로나-19는 아직 효과가 확인된 치료제가 없으므로 인플루엔자와 같이 환자를 대상으로 치료제를 사용하여 감염전파기간을 줄이는 방법은 사용할 수가 없다.

우리 모델링 결과는 무증상감염자에 의한 전파와 감염자 접촉에 의한 자가격리자들을 고려하지 않은 결과로, 무증상감염자에 의한 감염전파에 대한 영향과 이에 대한 관리효과, 자가격리자들의 관리효과 등은 포함되지 않았다. TANG, Biao와 그 외 연구진의 연구 결과에서 알 수 있듯이, 집중적인 접촉자 추적으로 인한 무증상감염자 관리와 자가격리는 감염재생산수를 줄이는데 효과적임을 알 수 있다 [8]. 방역당국에서는 2월 23일 우리나라 감염병 위기경보를 가장 높은 단계인 ‘심각’으로 격상하고 대구/경북 지역에 특별방역대책을 실행 중이다. 대구/경북 확진자 발생 대응을 위해 자가격리자 전담인력 확보 등의 자가격리자 이탈 방지를 위한 정책 뿐 만 아니라, 현재 코로나-19 감염전파의 소스가 된 신천지 대구교회 신도 명단을 확보하여 격리 및 검사를 실시 하고, 신천지 집회를 전면 금지하는 등의 방역정책을 실시하고 있어 근 시일내에 그 효과가 나타날 것이라 생각된다.n,

본 연구의 제한점은 첫째, 대구/경북의 모델링에서 환자들의 증상발생일 자료를 사용하지 못하고 확진일자 자료를 사용하였다는 것이다. 따라서 실제 환자 발생 양상에 대한 예측 일정은 연구결과보다 조금 이르게 나타날 가능성이 있다. 둘째, 증상발현에서 진단까지의 기간을 4일과 2일로 고정하여 분석하였기에 이로 인한 오차가 발생할 수 있다. 코로나-19 환자가 급증하면서, 정부의 적극적인 방역조치와 더불어 감염자 스스로 코로나 감염의 인식도 빨라져 증상발생 후 진단까지의 기간이 유행초기에는 길었다가 유행이 진행되면서 점차 빨라진 경향이 있기 때문이다. 하지만 개인별 차이가 있어 일률적으로 조정하지는 못하였다. 추후에 개인별 증상발생일과 진단일 자료를 보완한 모델이 필요하다. 셋째, 수학적 모델링을 단순화하기 위하여 잠복기 감염이나 무증상 감염을 고려하지 않았고, 환자들이 격리되면 감염 전파가 더 이상 일어나지 않는다고 가정하였다. 또한 이 모델에서는 해외에서 감염자가 들어오는 상황은 가정하지 않았고, 따라서 실제보다는 감염 환자 규모가 적게 추정되었을 가능성이 있으며 해외 유입상황에 따라 유행이 끝나지 않고 지속될 가능성이 있다. 대구/경북지역의 경우에도 미래에 추가적으로 대규모 집단감염이 발생하지 않을 것이라 가정하였기 때문에, 향후 추가적인 대규모 집단감염이 일어난다면, 유행양상은 모델의 결과와 달라질 수 있다. 마지막으로 한국 전체를 대상으로 모델링 하지 못하고 대구/경북을 위주로 분석한 것이다. 현재 한국 전체 유행의 약 90%를 대구/경북 유행이 차지하고 있어 나머지 지역과는 유행 양상이 매우 다를 것이어서 이 연구에서는 대구/경북 만을 대상으로 하였으나 향후 지역별 세부 모델링과 전국을 대상으로 한 모델링이 추가될 필요가 있다.

결론적으로, 한국의 초기 30일간의 유행은 약 30명의 환자가 발생하면서 $\mathcal{R}$ =0.5 수준으로 관리되었으나, 31번째 확진자부터 나타난 대구/경북의 유행은 $\mathcal{R}$ =3.5 수준으로 매우 빠르고 큰 규모로 나타났다. 하지만 방역당국의 적극적인 코로나-19검사로 환자들의 감염전파기간이 짧아지고, 국민들의 마스크 쓰기, 사회적 거리두기 등의 감염 예방조치 적극 참여 효과로 인하여 전파율이 낮아지고 있는 것으로 나타나고 있다. 유행의 규모를 줄이고 환자발생이 가능한 빨리 최저 수준으로 떨어져 총 유행기간을 줄이고 일상으로 돌아가기 위해서는 지속적인 방역당국의 노력과 함께 보다 적극적인 국민 참여 방역 운동이 필요하다. 특히 감염취약그룹이 모여 있는 집단 시설의 감염관리를 철저히 하여 국지적인 집단 유행이 발생하지 않도록 하여야 한다. 또한 중국을 제외한 전세계에서 유행이 증가하는 양상이므로 해외 유입 감염을 막기위한 입국자 대상 검역관리에도 지속적인 노력이 필요하다. 우리 감염병 모델링 연구팀은 계속 새로운 정보를 축적하여 모델링 연구 결과들을 수정하고 발전시켜 나갈 예정이다.

**Acknowledgement**

코로나-19 유행으로 방역과 치료에 애쓰고 있는 질병관리본부와 보건소 직원들, 현장에서 역학조사를 수행하고 있는 모든 역학조사관들, 전국의 의료진들께 깊이 감사드립니다. 코로나-19 유행을 종식시키기 위하여 개인의 불편을 감수하고 사회적 거리두기에 적극 참여해주시는 국민들께도 감사드립니다. 이 연구과제의 모델링 방법과 결과 해석에 대한 창의적인 제언을 아끼지 않고 해 주신 감염병 모델링 연구팀원들께도 감사드립니다.

This research was supported by Government-wide R&D Fund project for infectious disease research (GFID), Republic of Korea (grant No. HG18C0088).

This research was supported by the Korea National Research Foundation (NRF) grant funded by the Korean government (MEST) (NRF-2019R1A2B5B01101143).

**CONFLICT OF INTEREST**

The authors have no conflicts of interest to declare for this study.

**References**

1. Statement on the second meeting of the International Health Regulations (2005) Emergency Committee regarding the outbreak of novel coronavirus (2019-nCoV): The World Health Organization, 2020.

2. Korea Centers for Disease Control and Prevention. Current status of COVID-19 outbreak in Republic of Korea. Available from:

<http://ncov.mohw.go.kr/tcmBoardList.do?brdId=&brdGubun=&dataGubun=&ncvContSeq=&contSeq=&board_id=>.

3. Korean Statistical Infromation Service. Population Cencus. Available from:

<http://kosis.kr/statisticsList/statisticsListIndex.do?menuId=M_01_01&vwcd=MT_ZTITLE&parmTabId=M_01_01>

4. KI, Moran, et al. Epidemiologic characteristics of early cases with 2019-nCoV disease in Republic of Korea. Epidemiology and Health, 2020, e2020007.

5. Li Q, Guan X, Wu P, Wang X, Zhou L, Tong Y, et al. Early Transmission Dynamics in Wuhan, China, of Novel Coronavirus-Infected Pneumonia. N Engl J Med 2020.

6. WU, Joseph T.; LEUNG, Kathy; LEUNG, Gabriel M. Nowcasting and forecasting the potential domestic and international spread of the 2019-nCoV outbreak originating in Wuhan, China: a modelling study. The Lancet, 2020, 395.10225: 689-697.

7. ZHANG, Sheng, et al. Estimation of the reproductive number of Novel Coronavirus (COVID-19) and the probable outbreak size on the Diamond Princess cruise ship: A data-driven analysis. International Journal of Infectious Diseases, 2020.

8. TANG, Biao, et al. Estimation of the transmission risk of the 2019-nCoV and its implication for public health interventions. Journal of Clinical Medicine, 2020, 9.2: 462.

**Appendix**

App_Fig 1. 감염전파율과 감염전파기간의 변화에 따른 감염재생산수에 대한 등고선

App_Fig 2. 방역조치가 없을 때를 가정한 대구/경북 유행 모델링에 따른 확진일별 환자수.

: 피팅 기간에 따라 R이 변화함. (Base scenario)

App_Fig 3. 방역조치 효과가 나타나는 시점이 3월5일 부터이고 전파기간 4일, 전파율을 90%로 감소시켰을 때(Scenario 1), 3월5일 부터이고 전파기간 4일, 전파율을 99%로 감소시켰을 때(Scenario 2), 3월5일 부터이고 전파기간을 2일로 감소시키고, 전파율도 99% 감소시켰을 때 (Scenario 3), 2월29일 부터이고 전파기간 4일, 전파율을 90%로 감소시켰을 때(Scenario 4), 2월29일 부터이고 전파기간은 2일로 감소시키고, 전파율은 75%로 감소시켰을 때(Scenario 5) 대구/경북 유행 모델링에 따른 확진일별 환자수.


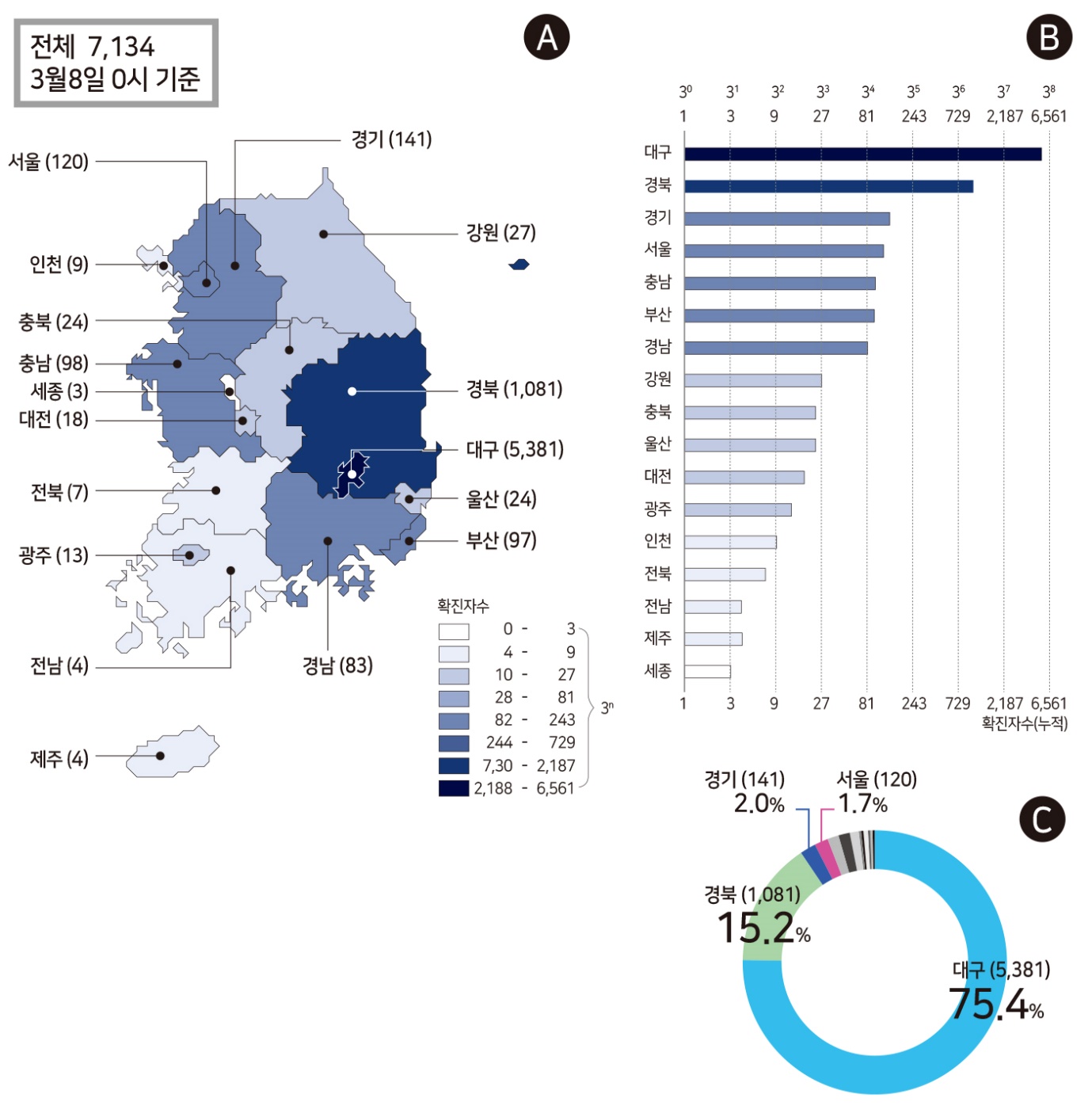


App_Fig 4. 3월8일까지 지역별 누적확진자수와 지역별 비율
