# Supplementary material for: Estimating the reproductive number and the outbreak size of COVID-19 in Korea
Source: Epidemiol Health. 2020 Mar 12;42:e2020011. doi: 10.4178/epih.e2020011 (PMC7285447; doi:10.4178/epih.e2020011)
Supplement: Supplementary file 2 [file epih-42-e2020011-app1.pdf]

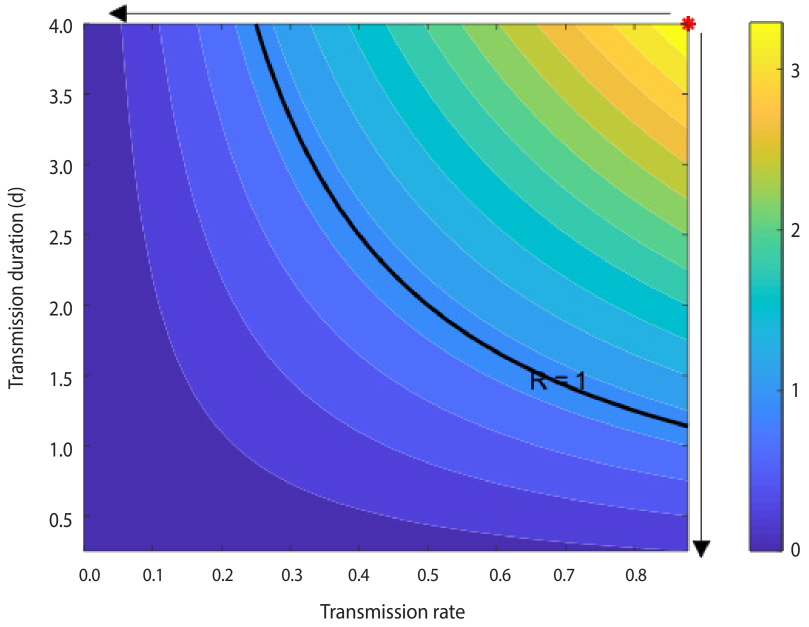

Appendix 1. A contour map of the reproductive number (*R*) as transmission rate and transmission duration changes. *R*=3.5 (red spot) and *R*=1 (black line).
