# Supplementary material for: Estimating the reproductive number and the outbreak size of COVID-19 in Korea
Source: Epidemiol Health. 2020 Mar 12;42:e2020011. doi: 10.4178/epih.e2020011 (PMC7285447; doi:10.4178/epih.e2020011)
Supplement: Supplementary file 3 [file epih-42-e2020011-app2.pdf]

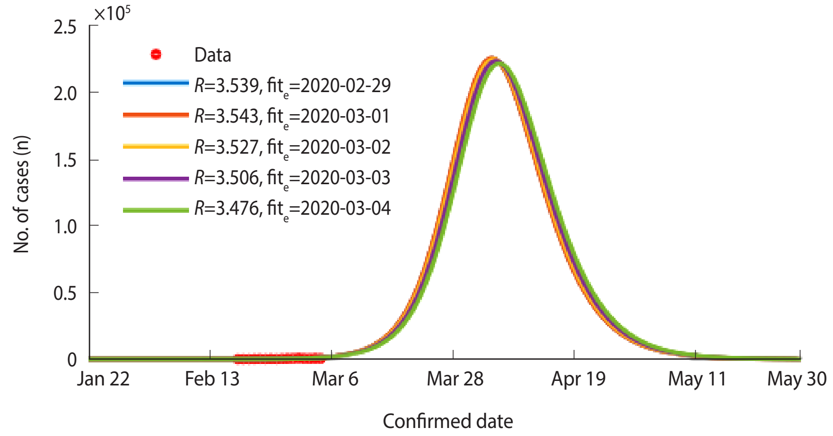

Appendix 2. Estimated reproductive number (*R*) and daily number of confirmed patients in Daegu and North Gyeongsang Province at base scenario which means no preventive measures. *R* varies with fitting period. Number of cases (red dots) and model fitting curves (colored lines).

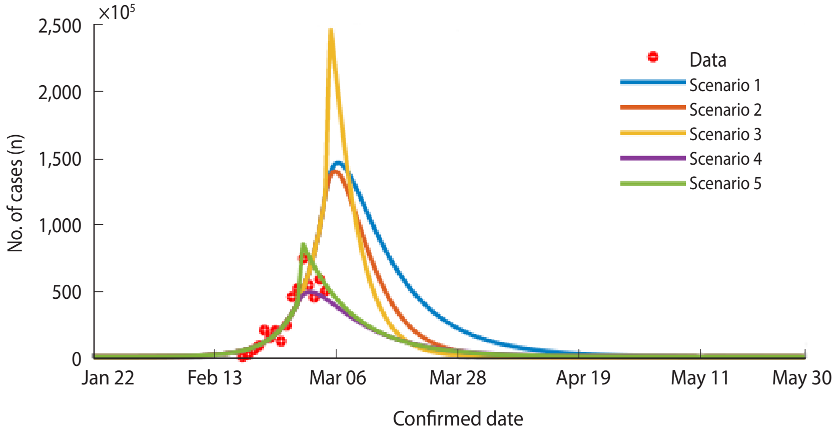

Appendix 3. Estimated daily number of confirmed patients by scenario in Daegu and North Gyeongsang Province. See Table 2 for scenarios. Number of cases (red dots) and model fitting curves (colored lines).
