# Supplementary material for: Estimating the reproductive number and the outbreak size of COVID-19 in Korea
Source: Epidemiol Health. 2020 Mar 12;42:e2020011. doi: 10.4178/epih.e2020011 (PMC7285447; doi:10.4178/epih.e2020011)
Supplement: Supplementary file 5 [file epih-42-e2020011-app4.pdf]

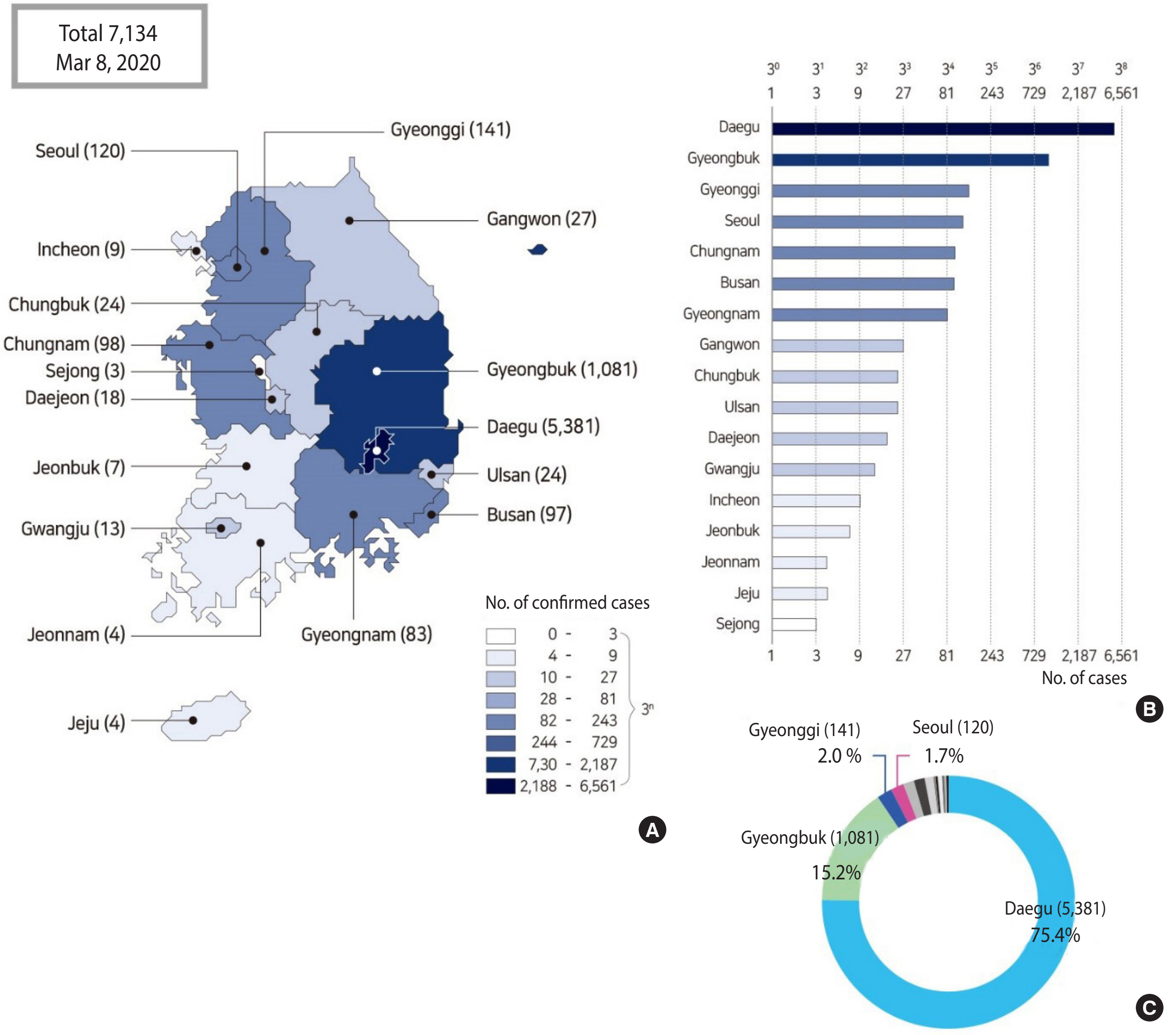

Appendix 4. Cumulative confirmed number of patients and ratio by region on March 8, 2020. (A) and (B) the national distribution of the cumulative confirmed cases in Korea; (C) the ratio of cumulative confirmed cases by region; Gyeongbuk is North Gyeongsang Province.
